# Supplementary material for: Antidepressant Use and Its Association with 28-Day Mortality in Inpatients with SARS-CoV-2: Support for the FIASMA Model against COVID-19
Source: J Clin Med. 2022 Oct 5;11(19):5882. doi: 10.3390/jcm11195882 (PMC9572995; doi:10.3390/jcm11195882)
Supplement: Supplementary file 1 [file jcm-11-05882-s001.zip › jcm-1935717-supplementary.pdf]

SUPPLEMENTARY MATERIAL

Figure S1. Prevalence of antidepressant use in inpatients hospitalized with and without COVID-19 by FIASMA activity (A), S1R affinity (B) and antidepressant class by FIASMA activity (C-D) (N = 82,586).

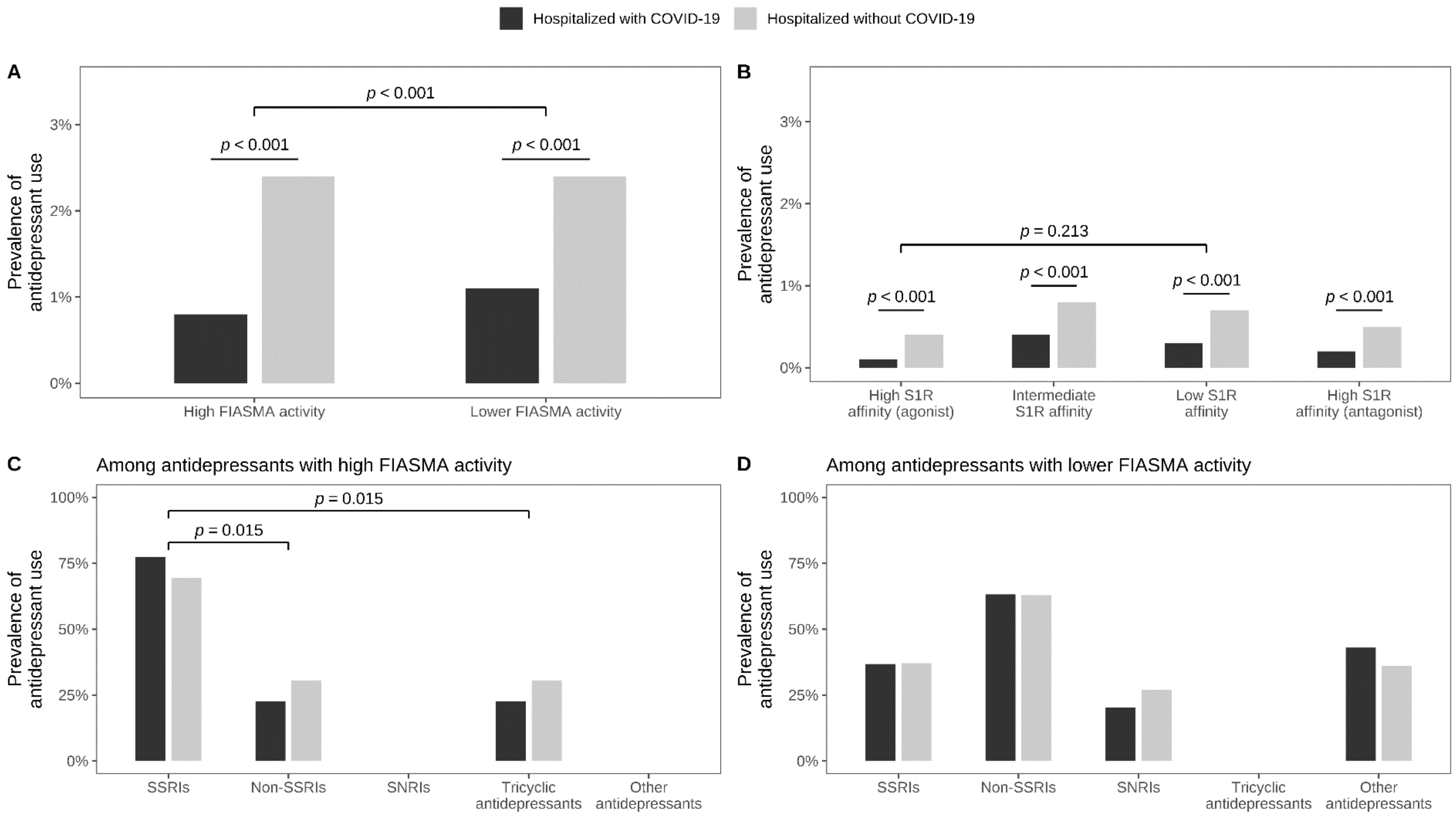

Figure S2. Antidepressant use and 28-day all-cause mortality in a matched analytic sample of patients hospitalized with COVID-19 by FIASMA activity (A), S1R affinity (B) and antidepressant class by FIASMA activity (C-D) (N=1,482).

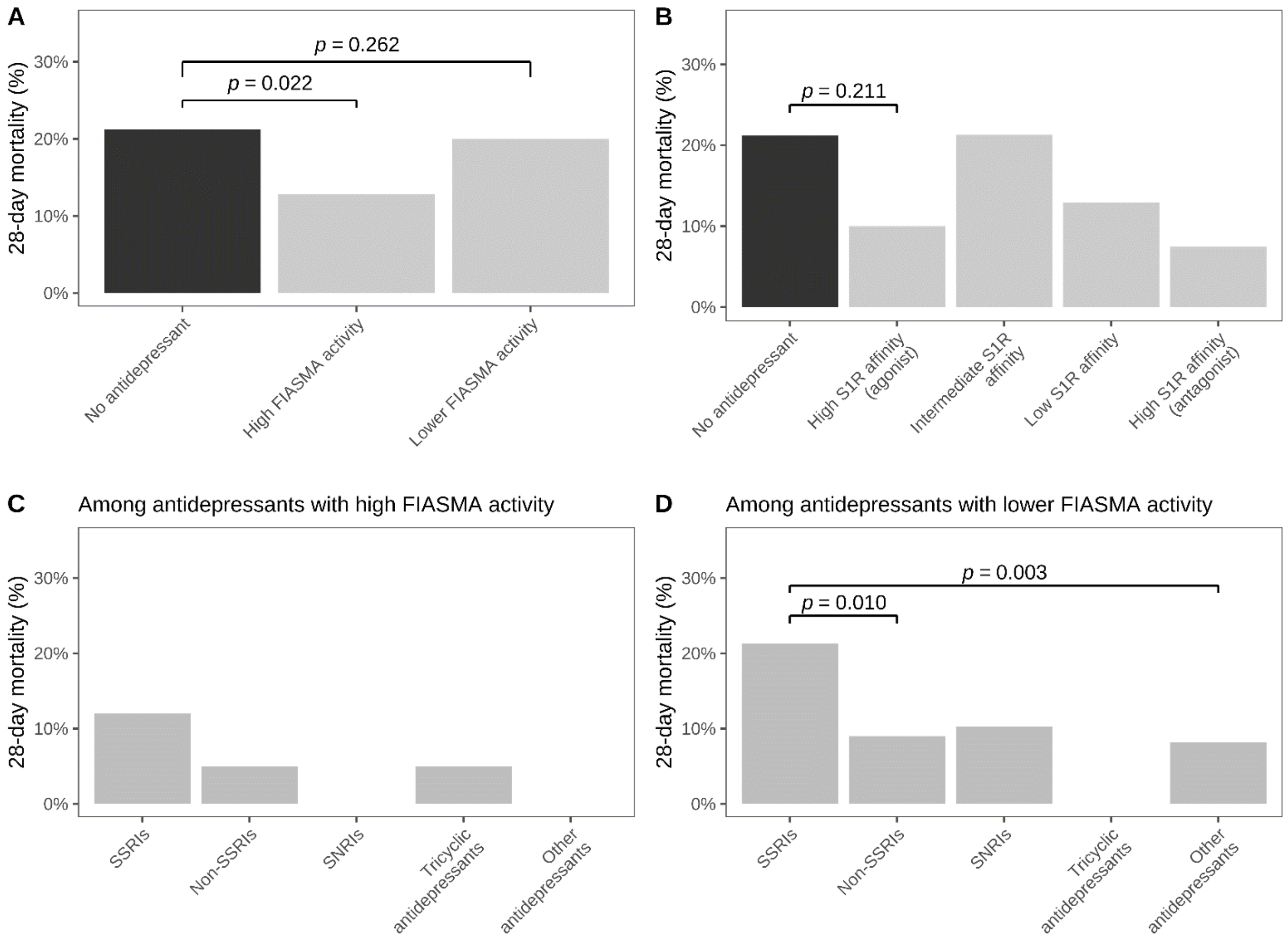

Table S1. Comparing characteristics between patients hospitalized with COVID-19 and patients hospitalized without COVID-19 in a 1:1 ratio matched analytic sample (N=82,586).

	With COVID-19 (N=41,293)	Without COVID-19 (N=41,293)	With COVID-19 vs. without COVID-19
	Median (SD) / N (%)	Median (SD) / N (%)	SMD
Age	53.7 (21.4)	53.6 (21.6)	0.003
Sex			<0.001
Women	20561 (50.0%)	20552 (50.0%)	
Men	20732 (50.0%)	20741 (50.0%)	
Hospital			0.006
AP-HP Centre—Paris University, Paris Saclay University, Henri Mondor University Hospitals and at home hospitalization and Hôpitaux	17192 (50.1%)	17136 (49.9%)	
AP-HP Nord and Universitaires Paris Seine-Saint-Denis	16454 (50.1%)	16418 (49.9%)	
AP-HP Sorbonne University	7647 (49.7%)	7739 (50.3%)	
Hospitalization period			0.003
May 2nd 2020 - December 5th 2020	16195 (50.0%)	16224 (50.0%)	
December 6th 2020 - March 15th 2021	9598 (49.9%)	9629 (50.1%)	
March 16th 2021 - November 2nd 2021	15500 (50.1%)	15440 (49.9%)	
Number of medical conditions			0.023
0-4	29155 (50.1%)	29089 (49.9%)	
5-7	4397 (49.7%)	4443 (50.3%)	
8-9	2284 (49.2%)	2362 (50.8%)	
10-12	2409 (49.5%)	2454 (50.5%)	
13-17	2049 (49.7%)	2073 (50.3%)	
18+	999 (53.4%)	872 (46.6%)	

SMD>0.1 indicate substantial differences.
Abbreviation: SMD, standardized mean difference.

Table S2. Prevalence of antidepressant use in a matched analytic sample of adult patients hospitalized with and without COVID-19 (A), and antidepressant use and 28-day all-cause mortality in a matched analytic sample of patients hospitalized with COVID-19 (B), using the nearest neighbor matching method.

**A. Matched analytic sample of adult patients hospitalized with and without COVID-19**	**Patients hospitalized with COVID-19** (N = 41,293) – N (%)	**Patients hospitalized without COVID-19** (N = 41,293) – N (%)	**Hospitalized with COVID-19 *versus* without COVID-19 in a 1:1 ratio matched analytic sample** – OR (95%CI; p-value)	**Hospitalized with COVID-19 *versus* without COVID-19 in a 1:1 ratio matched analytic sample adjusted for unbalanced covariates** – AOR (95%CI; p-value)
No antidepressant	40521 (98.1%)	39259 (95.1%)	Ref.	Ref.
Any antidepressant	772 (1.9%)	2035 (4.9%)	0.37 (0.34 - 0.40; <0.001***)	-
**B. Matched analytic sample of patients hospitalized with COVID-19**	**Median dose (IQR)**	**Deaths / Patients (%)**	**Crude logistic regression in the matched analytic sample** – OR (95%CI; p-value)	**Multivariable logistic regression in the matched analytic sample adjusted for unbalanced covariates** – AOR (95%CI; p-value)
No antidepressant	-	196 / 741 (26.5%)	Ref.	Ref.
Any antidepressant	30.0 (19.0-49.5)	95 / 741 (12.8%)	0.41 (0.31 - 0.54; <0.001***)	0.42 (0.32 - 0.55; <0.001***)[a]

[a] Adjusted for age, number of medical conditions and any antipsychotic medication.
* Two-sided p < 0.05; ** p < 0.01; *** p < 0.001.
Abbreviations: OR, odds ratio; AOR, adjusted odds ratio; CI, confidence interval; NA, not applicable; -, irrelevant.

Table S3. Associations of baseline characteristics with 28-day mortality in the matched analytic sample of inpatients with COVID-19 receiving or not receiving an antidepressant at baseline (N=1,482).

	Full population (N=1,482)	Death (N=252)	No death (N=1230)	Crude analysis	Multivariable analysis	
	Mean (SD)/ N (%)	Mean (SD)/ N (%)	Mean (SD)/ N (%)	OR (95%CI ; p-value)	AOR (95%CI ; p-value)	Collinearity diagnosis (GVIF)
Age	76.2 (15.4)	82.5 (9.76)	75.0 (16.0)	1.87 (1.57 - 2.24; <0.001*)	1.06 (1.05 - 1.08; <0.001*)	1.24
Sex						1.09
Women	931 (62.8%)	145 (15.6%)	786 (84.4%)	Ref.	Ref.	
Men	551 (37.2%)	107 (19.4%)	444 (80.6%)	1.31 (0.99 - 1.72; 0.057)	1.67 (1.22 - 2.29; 0.001*)	
Hospital						1.23
AP-HP Centre—Paris University, Paris Saclay University, Henri Mondor University Hospitals and at home hospitalization and Hôpitaux	626 (42.2%)	100 (16.0%)	526 (84.0%)	Ref.	Ref.	
AP-HP Nord and Universitaires Paris Seine-Saint-Denis	373 (25.2%)	64 (17.2%)	309 (82.8%)	1.09 (0.77 - 1.54; 0.625)	1.11 (0.75 - 1.66; 0.592)	
AP-HP Sorbonne University	483 (32.6%)	88 (18.2%)	395 (81.8%)	1.17 (0.86 - 1.61; 0.323)	1.10 (0.77 - 1.58; 0.602)	
Hospitalization period						1.08
May 2nd 2020 - December 5th 2020	519 (35.0%)	69 (13.3%)	450 (86.7%)	Ref.	Ref.	
December 6th 2020 - March 15th 2021	497 (33.5%)	101 (20.3%)	396 (79.7%)	1.66 (1.19 - 2.32; 0.003*)	1.53 (1.06 - 2.21; 0.023*)	
March 16th 2021 - November 2nd 2021	466 (31.4%)	82 (17.6%)	384 (82.4%)	1.39 (0.98 - 1.97; 0.062)	1.53 (1.04 - 2.24; 0.030*)	
Number of medical conditions						1.45
0-4	268 (18.1%)	35 (13.1%)	233 (86.9%)	Ref.	Ref.	
5-7	316 (21.3%)	45 (14.2%)	271 (85.8%)	1.11 (0.69 - 1.78; 0.679)	0.94 (0.55 - 1.60; 0.826)	
8-9	206 (13.9%)	24 (11.7%)	182 (88.3%)	0.88 (0.50 - 1.53; 0.645)	0.66 (0.35 - 1.22; 0.182)	
10-12	239 (16.1%)	47 (19.7%)	192 (80.3%)	1.63 (1.01 - 2.63; 0.045*)	1.49 (0.86 - 2.59; 0.156)	
13-17	303 (20.4%)	66 (21.8%)	237 (78.2%)	1.85 (1.18 - 2.90; 0.007*)	1.30 (0.77 - 2.21; 0.327)	
18+	150 (10.1%)	35 (23.3%)	115 (76.7%)	2.03 (1.21 - 3.40; 0.008*)	1.43 (0.77 - 2.64; 0.258)	
Any psychiatric disorder [1]						1.36
Yes	775 (52.3%)	137 (17.7%)	638 (82.3%)	1.11 (0.84 - 1.45; 0.470)	0.79 (0.56 - 1.12; 0.181)	
No	707 (47.7%)	115 (16.3%)	592 (83.7%)	Ref.	Ref.	
Any medication according to						1.21

compassionate use or as part of a medical trial						
Yes	298 (20.1%)	39 (13.1%)	259 (86.9%)	4.03 (3.00 - 5.42; <0.001*)	3.75 (2.67 - 5.26; <0.001*)	
No	1184 (79.9%)	213 (18.0%)	971 (82.0%)	Ref.	Ref.	
Any benzodiazepine or Z-drug within 24h from admission						1.11
Yes	644 (43.5%)	179 (27.8%)	465 (72.2%)	1.51 (1.15 - 1.99; 0.003*)	1.64 (1.20 - 2.24; 0.002*)	
No	838 (56.5%)	73 (8.71%)	765 (91.3%)	Ref.	Ref.	
Any antipsychotic medication within 24h from admission						1.08
Yes	781 (52.7%)	154 (19.7%)	627 (80.3%)	0.91 (0.64 - 1.30; 0.603)	1.17 (0.78 - 1.74; 0.449)	
No	701 (47.3%)	98 (14.0%)	603 (86.0%)	Ref.	Ref.	
Any mood stabilizer medication within 24h from admission						1.02
Yes	282 (19.0%)	45 (16.0%)	237 (84.0%)	0.69 (0.48 - 0.99; 0.045*)	0.84 (0.56 - 1.26; 0.400)	
No	1200 (81.0%)	207 (17.2%)	993 (82.8%)	Ref.	Ref.	
Biological severity of COVID-19 [2]						1.25
Yes	782 (52.8%)	168 (21.5%)	614 (78.5%)	2.01 (1.51 - 2.67; <0.001*)	1.3 (0.93 - 1.84; 0.129)	
No	700 (47.2%)	84 (12.0%)	616 (88.0%)	Ref.	Ref.	
Clinical severity of COVID-19 [3]						1.11
Yes	540 (36.4%)	128 (23.7%)	412 (76.3%)	2.05 (1.56 - 2.70; <0.001*)	1.62 (1.18 - 2.2; 0.002*)	
No	942 (63.6%)	124 (13.2%)	818 (86.8%)	Ref.	Ref.	

[1] Assessed using ICD-10 diagnosis codes for any psychiatric disorder (F00-F99).
[2] Defined as having at least one of the following criteria: high neutrophil-to-lymphocyte ratio or low lymphocyte-to-C-reactive protein ratio (both variables were dichotomized at the median of the values observed in the full sample), or plasma lactate levels higher than 2 mmol/L.
[3] Defined as having at least one of the following criteria: respiratory rate > 24 breaths/min or < 12 breaths/min, resting peripheral capillary oxygen saturation in ambient air < 90% , temperature > 40°C, or systolic blood pressure < 100 mm Hg.
* Two-sided p-value is significant (p<0.05).
Abbreviations: OR, odds ratio; AOR, adjusted odds ratio; CI, confidence interval; GVIF, generalized variance inflation factor; NA, not applicable.

Table S4. Characteristics of patients receiving or not receiving an antidepressant at baseline in the full sample and in the 1:1 ratio matched analytic sample of patients hospitalized with COVID-19.

	With any antidepressant (N=741)	Without antidepressants in the full sample (N=40,521)	Without antidepressants in the matched control group (N=741)	Any antidepressant vs. without antidepressants in the full sample	Any antidepressant vs. without antidepressants in the matched analytic sample
	Mean (SD)/N (%)	Mean (SD)/N (%)	Mean (SD)/N (%)		SMD
Age	77.0 (15.2)	53.2 (21.2)	75.5 (15.5)	1.288	0.099
Sex				0.303	0.064
Women	477 (64.4%)	20065 (49.5%)	454 (61.3%)		
Men	264 (35.6%)	20456 (50.5%)	287 (38.7%)		
Hospital				0.403	0.036
AP-HP Centre—Paris University, Paris Saclay University, Henri Mondor University Hospitals and at home hospitalization and Hôpitaux	307 (41.4%)	16855 (41.6%)	319 (43.0%)		
AP-HP Nord and Universitaires Paris Seine-Saint-Denis	187 (25.2%)	16267 (40.1%)	186 (25.1%)		
AP-HP Sorbonne University	247 (33.3%)	7399 (18.3%)	236 (31.8%)		
Hospitalization period				0.252	0.065
May 2nd 2020 - December 5th 2020	267 (36.0%)	15910 (39.3%)	252 (34.0%)		
December 6th 2020 - March 15th 2021	252 (34.0%)	9334 (23.0%)	245 (33.1%)		
March 16th 2021 - November 2nd 2021	222 (30.0%)	15277 (37.7%)	244 (32.9%)		
Number of medical conditions				1.322	0.038
0-4	129 (17.4%)	29003 (71.6%)	139 (18.8%)		
5-7	161 (21.7%)	4232 (10.4%)	155 (20.9%)		
8-9	103 (13.9%)	2178 (5.37%)	103 (13.9%)		
10-12	120 (16.2%)	2289 (5.65%)	119 (16.1%)		
13-17	153 (20.6%)	1895 (4.68%)	150 (20.2%)		
18+	75 (10.1%)	924 (2.28%)	75 (10.1%)		
Any psychiatric disorder [1]				1.122	0.003
Yes	388 (52.4%)	3059 (7.55%)	387 (52.2%)		
No	353 (47.6%)	37462 (92.5%)	354 (47.8%)		
Any medication according to compassionate use or as part of a medical trial				0.696	0.027

Yes	317 (42.8%)	5375 (13.3%)	327 (44.1%)		
No	424 (57.2%)	35146 (86.7%)	414 (55.9%)		
Any benzodiazepine or Z-drug within 24h from admission				1.286	0.003
Yes	391 (52.8%)	1618 (3.99%)	390 (52.6%)		
No	350 (47.2%)	38903 (96.0%)	351 (47.4%)		
Any antipsychotic medication within 24h from admission				0.633	<0.001
Yes	141 (19.0%)	379 (0.94%)	141 (19.0%)		
No	600 (81.0%)	40142 (99.1%)	600 (81.0%)		
Any mood stabilizer medication within 24h from admission				0.643	<0.001
Yes	149 (20.1%)	496 (1.22%)	149 (20.1%)		
No	592 (79.9%)	40025 (98.8%)	592 (79.9%)		
Biological severity of COVID-19 [2]				0.407	0.022
Yes	387 (52.2%)	13168 (32.5%)	395 (53.3%)		
No	354 (47.8%)	27353 (67.5%)	346 (46.7%)		
Clinical severity of COVID-19 [3]				0.527	0.006
Yes	271 (36.6%)	5817 (14.4%)	269 (36.3%)		
No	470 (63.4%)	34704 (85.6%)	472 (63.7%)		

[1] Assessed using ICD-10 diagnosis codes for any psychiatric disorder (F00-F99).
[2] Defined as having at least one of the following criteria: high neutrophil-to-lymphocyte ratio or low lymphocyte-to-C-reactive protein ratio (both variables were dichotomized at the median of the values observed in the full sample), or plasma lactate levels higher than 2 mmol/L.
[3] Defined as having at least one of the following criteria: respiratory rate > 24 breaths/min or < 12 breaths/min, resting peripheral capillary oxygen saturation in ambient air < 90% , temperature > 40°C, or systolic blood pressure < 100 mm Hg.
SMD>0.1 indicate substantial differences.
Abbreviation: SMD, standardized mean difference.

Table S5. Association of antidepressant use with urinary infection in the matched analytic sample of inpatients with COVID-19 receiving or not receiving an antidepressant at baseline (N=1,482).

	**Number of events / Number of patients (%)**	**Crude logistic regression**
	**N / N (%)**	**OR (95%CI; p-value)**
**Full sample**		
Without antidepressants	31 / 741 (4.2%)	Ref.
Any antidepressant	38 / 741 (5.1%)	1.24 (0.76 - 2.01; 0.389)

Urinary infection was defined as having any ICD-10 code for acute pyelonephritis (N10) or cystitis (N30).
Abbreviations: OR, odds ratio; CI, confidence interval.
